# A Simplified Method for Predicting Bond–Slip Behaviour of Ribbed Bars and Threaded Rods Glued in Glulam along the Grain

**DOI:** 10.3390/ma16155370

**Published:** 2023-07-31

**Authors:** Jun Peng, Dan-Dan Wang, Shao-Bo Kang, Shu-Rong Zhou

**Affiliations:** 1 Department of Civil Engineering, Chongqing Vocational Institute of Engineering, Chongqing 402260, China; pengjunxiansheng@163.com; 2 School of Civil Engineering, Chongqing University, Chongqing 400045, China; wangdd0914@cqu.edu.cn (D.-D.W.); kang0119@cqu.edu.cn (S.-B.K.)

**Keywords:** bond strength, ribbed bars, steel rods, glulam, analytical method, design equation

## Abstract

The bond between a steel reinforcement/rod and glulam plays a crucial role in the resistance and deformation capacity of timbers joints. Existing studies provide different bond–slip models for reinforcements and rods with different anchorage lengths, in which the relationship between local bond stress and global bond behaviour cannot not be established. This study presents a unified analytical method for predicting the bond–slip behaviour of ribbed bars and threaded rods along the grain using a local bond–slip model of reinforcement at the elastic and post-yield stages. In the analytical method, equilibrium, compatibility, and constitutive models for reinforcement and rods are considered. The method is verified using test data of rebars and rods with different anchorage lengths. Comparisons between the experimental and calculated results suggest that the analytical method yields reasonably good predictions of the load–slip relationship and failure mode. Furthermore, the embedment lengths required for yield and the ultimate strengths of the reinforcement and rods along the grain are determined by assuming uniform bond stress distributions over the elastic and post-yield steel segment. The average bond stress over the entire anchorage length is calculated and compared with existing equations. Design recommendations for anchorage lengths are proposed for ribbed bars and threaded rods glued in glulam.

## 1. Introduction

Glued-in rods have been widely used for column foundations and moment frames in timber structures due to their high resistance and stiffness [1,2]. In the design of connections with glued-in rods, the anchorage length and bond strength of rods play a critical role in determining the resistance and ductility of the connection.

Various methods have been proposed to calculate the pull-out resistance of steel rods with different lengths glued in glulam [3,4,5]. In general, the bond stress can be assumed to be uniformly distributed along the anchorage length, and the resistance of rods along the grain can be determined using the average bond strength. The factored average bond strength can also be used to take account of the nonuniform bond stress distribution over a long anchorage length. The influence of the number and spacing of rods on the pull-out resistance of joints with multiple rods was also determined in [6]. Xu et al. [7] investigated the influence of manufacturing defects on the pull-out behaviour of rods through experimental tests and numerical simulations, and concluded that failure was initiated at the end of the borehole due to the presence of high shear and tensile stresses.

Steel rods may remain elastic when designed with a practical range of anchorage length as a result of the high yield strength, unless the cross-section of the rods is intentionally reduced by removing the thread [4]. Pull-out tests on glued-in rebars [8,9] showed that ribbed bars developed inelastic behaviour under pull-out loads when the anchorage length exceeded 160 mm (10 times the rebar diameter), even though pull-out failure might still occur. Different bond–slip models were proposed for ribbed bars with specific anchorage lengths [8,9]. However, if the length of the reinforcement varies, it is not possible to examine whether the reinforcement yields or not. Existing test data on the bond strength of reinforcement embedded in concrete suggest that bond stress could be substantially reduced upon yielding of the reinforcement [10,11]. Similar phenomena could be expected for reinforcement glued in glulam. Thus, a unified method is needed to evaluate the bond–slip behaviour of reinforcement with different anchorage lengths, through which the stress state and failure model of the anchored reinforcement along the grain can be determined in a straightforward manner. 

This study presents a simplified method for evaluating the bond–slip behaviour of reinforcement glued in glulam along the grain. In our method, the relationship between a local bond–slip model of short reinforcement and the global response of long reinforcement is established, and the bond stresses of ribbed bars and threaded rods at the elastic and post-yield stages are considered. The force–slip relationship of the reinforcement is obtained via an analytical study, and the variations in steel strain and bond stress over the length of the reinforcement are also calculated. The analytical results show the profiles of steel strain and bond stress at various load levels, which enable the development of a design equation for quantifying the average bond strength. With the average bond strength, the lengths of glued-in rebars and rods required for the rebars and rods to reach the yield and ultimate strengths are calculated.

## 2. Bond–Slip Models

**Figure 1:**
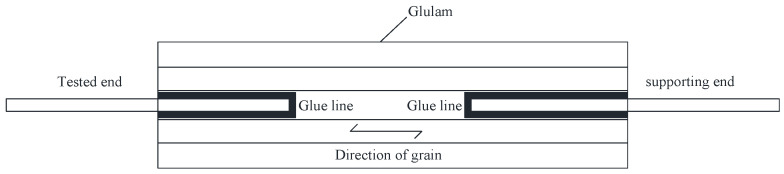
Pull-out tests for glued-in rebars or rods.

Pull-out tests can be performed to obtain a bond–slip model of steel glued in glulam. Different loading methods are recommended for pull-out tests [12], among which pull–pull loading is often adopted for quantifying the bond strength of ribbed bars or threaded rods parallel to the grain of glulam, as shown in Figure 1. During testing, a tension force was applied at the tested end and the slip of the rebars or rods relative to the timber block was measured. A supporting end was also designed so that the specimen could be fixed to the testing machine.

The test results [4,9] showed that the bond strength of rebars or rods depended on several parameters, such as anchorage length, yield and ultimate strengths, the edge distance of the rebars or rods, the density of the timber, etc. In general, when the anchorage length of rebars or rods is relatively short, constant bond stress can be assumed over the length; otherwise, nonuniform bond stresses can be expected with the maximum value at the loaded end and the minimum value at the free end. When the yielding of reinforcement occurs near the loaded end, the associated bond stress drops rapidly due to inelastic elongation of the reinforcement. As a result, the assumption of constant bond stress is not valid. Indeed, the global bond–slip behaviour of long reinforcement can be calculated by dividing the reinforcement into a series of short steel segment and using a local bond–slip model for each segment, in which bond stresses at the elastic and post-yield stages can be considered along the anchorage length.

### 2.1. Local Bond–Slip Curve

**Figure 2:**
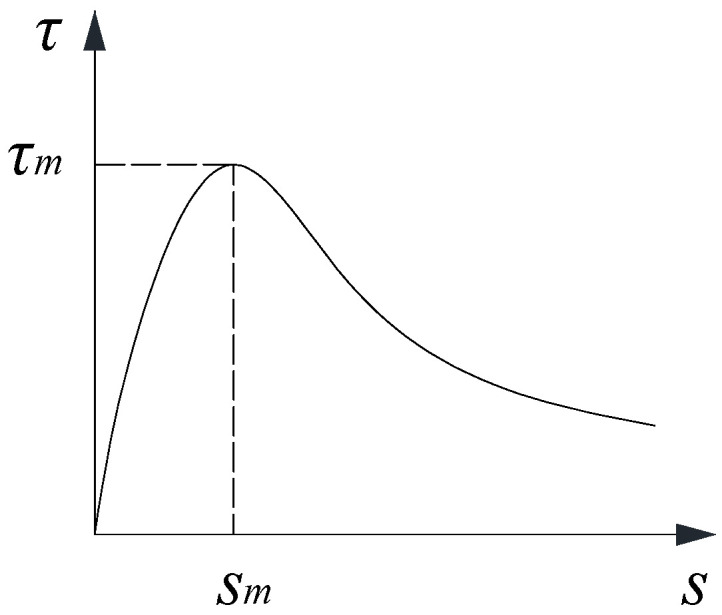
Local bond–slip model for glued-in rebars.

Ling et al. [8] conducted a series of pull-out tests on the bond strength of deformed bars glued in glulam. In the experimental programme, steel rebars with different anchorage lengths were tested, and a local bond–slip model was proposed for short rebars based on test data, as shown in Figure 2.

Equation (1) expresses the local bond–slip relationship. Note that the bond stress in the equation acts on the reinforcement, and the slip refers to the total value of deformation of the reinforcement, the glulam, and the adhesive between the reinforcement and glulam. In this study, the peak bond stress for rebars is set to be 9.1 MPa, taken from pull-out tests of short rebars anchored in glulam, and the values of α and β are 0.55 and 0.4, respectively. For glued-in steel rods, the bond strength can be taken as 10 MPa and is 0.75. As sudden pull-out failure of the threaded rods occurs, the descending branch should not be defined.

(1) $$\begin{array}{cc} \frac{\tau }{\tau_{m}}=\frac{1}{\alpha }{\left(\frac{s}{s_{m}}\right)}^{\alpha }-\;\left(\frac{1}{\alpha }\;-\;1\right)\frac{s}{s_{m}} & 0\;\leq \;s\;\leq \;s_{m}\\ \frac{\tau }{\tau_{m}}=\frac{s}{s_{m}}\frac{1}{\frac{s}{s_{m}}+\beta {\left(\frac{s}{s_{m}}-\;1\right)}^{2}} & s_{m}\;<\;s \end{array}$$

where $\tau_{m}$ is the peak bond stress, $s_{m}$ is the slip of the reinforcement at peak bond stress, and α and β are parameters defining the degradation of bond stress.

**Figure 3:**
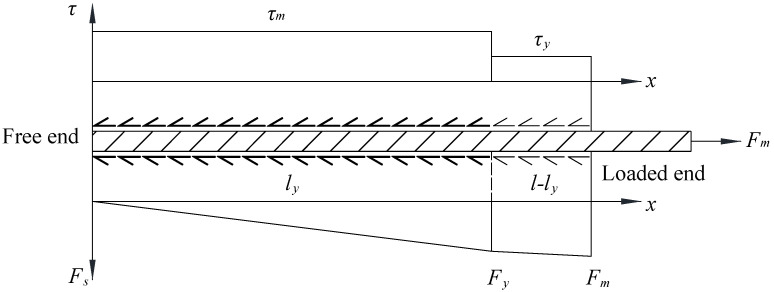
Bond stress distribution over inelastic steel reinforcement.

It should be pointed out that the foregoing equation is only valid for reinforcement at the elastic stage. If the anchorage length of the reinforcement is adequately long so that its yield strength can be reached at the loaded end, a different value of bond stress has to be adopted for the yielded steel segment. Previous studies on steel reinforcement embedded in concrete showed that bond stress remained nearly uniform over the yielded steel segment [10]. However, it is difficult to measure the post-yield bond stress through experimental tests. Analytical studies on bond stress distribution [13] showed that if steel reinforcement exhibited pull-out failure at the post-yield stage, bond stresses at the load capacity remained nearly constant over the elastic and post-yield steel segments. Moreover, the bond stress along the elastic steel segment reached peak bond stress. Therefore, post-yield bond stress can be calculated from the equilibrium of the ribbed bars or the threaded rods at the load capacity, as shown in Figure 3.

Equation (2) shows the expression for the post-yield bond stress. In the equation, $F_{m}$ lies between the yield and ultimate forces of the reinforcement and can be obtained through pull-out tests.

(2) $$\tau_{y}=\frac{F_{m}-\;F_{y}}{\pi d\left(l\;-\;l_{y}\right)}$$

where $\tau_{y}$ is the post-yield bond stress, $F_{m}$ is the maximum force of the rebars or rods, $F_{y}$ is the yield force of the rebars or rods, d is the diameter of the reinforcement, l is the anchorage length of the reinforcement, and $l_{y}$ the distance from the free end to the section where the reinforcement yields, and can be calculated using Equation (3).

(3) $$l_{y}=\frac{F_{y}}{\tau_{m}\pi d}$$

By using the abovementioned method, the post-yield bond strength of rebars or rods glued in glulam can be determined as $0.7\tau_{m}$ from the yield and ultimate loads of S16-200-2 [8]. Similar to rebars embedded in concrete [10], the reduction in bond strength after the yielding of reinforcement is mainly induced by inelastic elongation of the reinforcement.

### 2.2. Global Force–Slip Relationship

**Figure 4:**
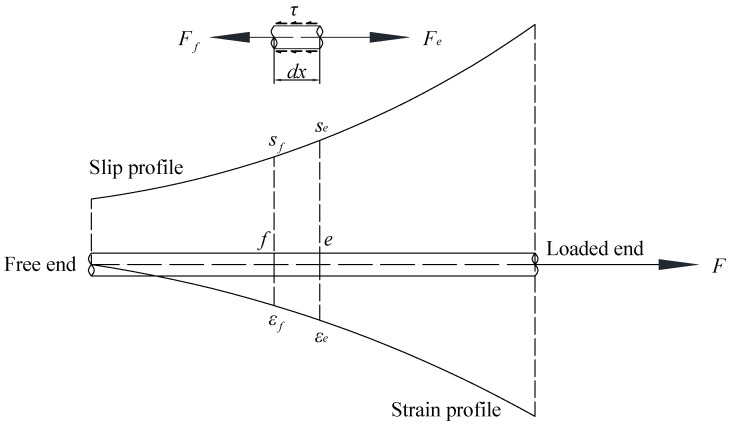
Equilibrium and compatibility of steel segment along the anchorage length.

**Figure 5:**
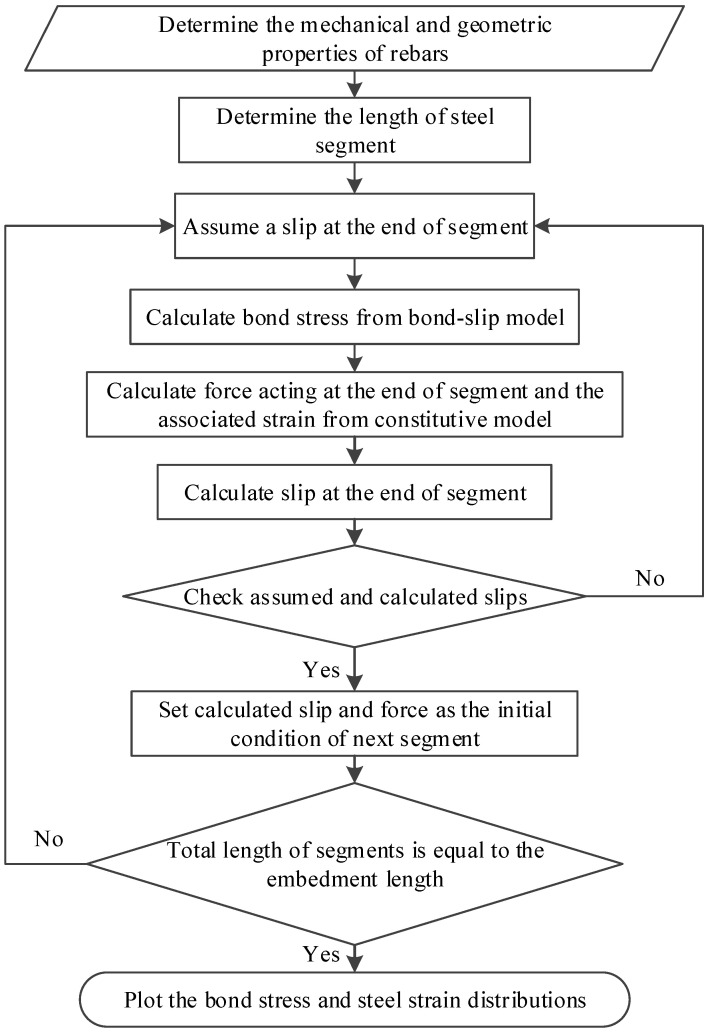
Solution procedures for glued-in rebars or rods with long anchorage lengths.

With the local bond–slip relationship, the bond–slip behaviour of long rebars or rods can be analysed by dividing the whole anchorage length into a group of steel segments, as shown in Figure 4, so that the bond stress over each steel segment can still be assumed to be uniform. Equations (4) and (5) shows the equilibrium and compatibility. It can be observed that the difference in forces at the two ends of the steel segment is equal to the bond force acting along the segment, and the difference in slips is the cumulative strain over the segment. By considering the equilibrium and compatibility for the steel segments from the free end to the loaded end, the profiles of bond stress, steel strain, and slip can be obtained by following the solution procedure presented in Figure 5. The analysis starts from the free end where the strain of the reinforcement or rods is zero. For a given slip at one end of the segment, the bond stress over the segment can be calculated from the proposed bond–slip model by assuming slip at the end of the segment. Thereafter, the slip at the other end of the segment can be calculated from the compatibility, and the stress can be computed from the equilibrium. Comparisons between the assumed and calculated slips should be made. If the difference falls within the prescribed tolerance, the slip, strain, and stress at the other end of the segment are determined and should be used as the boundary conditions of the next segment. The same procedure is repeated until the slip, strain, and stress over the whole reinforcement or the rods are quantified. Then, the load, slip, and strain at the loaded end of the reinforcement can be obtained from the study.

(4) $$F_{e}-\;F_{f}=\pi d\tau \mathrm{dx},$$

(5) $$s_{e}-\;s_{f}=\frac{1}{2}\left(\varepsilon_{e}+\varepsilon_{f}\right)\mathrm{dx}$$

where $F_{e}$ and $F_{f}$ are the forces acting at the ends of each segment, d is the diameter of the steel reinforcement, dx is the length of the steel segment, $s_{e}$ and $s_{f}$ are the slips at the end of the segment, and $\varepsilon_{e}$ and $\varepsilon_{f}$ are the strains at the end of the segment.

## 3. Verification of Local Bond–Slip Models

Most test data only provided the load capacity of glued-in rebars or rods under pull-out forces, and a limited number of load–slip curves were included in the literature. Ling et al. [8,9] conducted a series of pull-out tests on rebars and rods glued in glulam. In the design of specimens, two-component epoxy resin was used to glue the steel bars or the rod to the glulam, and the thickness of the glue line remained at 2 mm. Detailed information can be found in the references. Table 1 summarises the mechanical properties of the rebars or rods. Douglas fir with a density of 490 kg/m^3^ was used in the specimens, and its mechanical properties parallel to the grain can be found in Table 2.

It should be pointed out that only pull-out failure or fracture of the rebars or rods can be considered in the analytical method, and thus, rebars and rods with the two failure modes were selected from the experimental results and used to verify the accuracy of the proposed analytical method. The force–slip relationship of reinforcement glued in the glulam was calculated using the proposed method. Note that during calculation, the deformation of the glulam itself was neglected.

### 3.1. Bond–Slip Behaviour of Ribbed Bars

**Figure 6:**
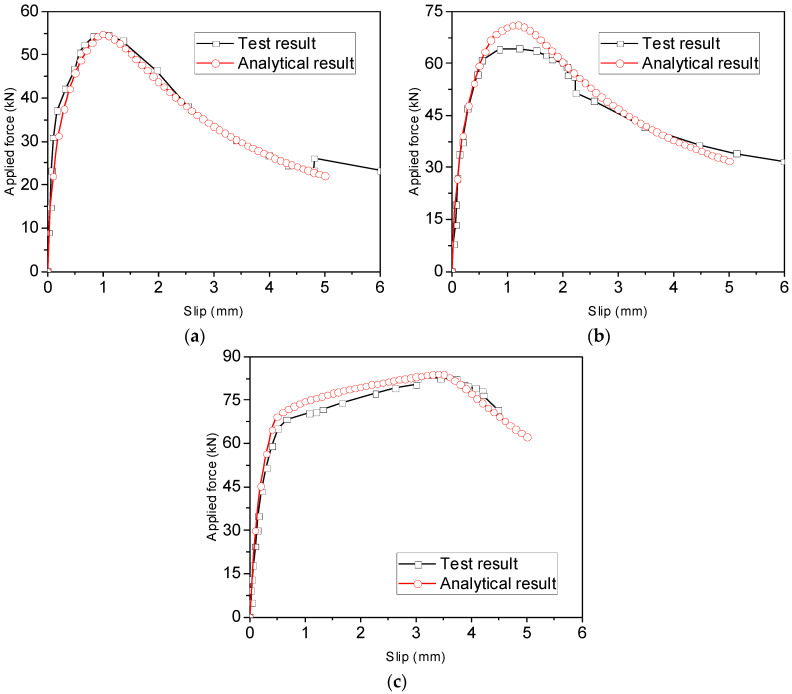
Comparison between experimental and analytical force–slip relationships of ribbed bars with different anchorage lengths [8] for (**a**) S16-120-2; (**b**) S16-160-2; and (**c**) S16-200-2.

Figure 6 shows a comparison between the analytical and experimental force–slip relationships of ribbed bars tested by Ling et al. [8]. The test results in the figure represent the average values of five specimens with or without grooving, and their cross-sectional areas of reinforcement differed from each other. It can be observed that the analytical results are in good agreement with the test results. The initial ascending stage, the plateau and the descending branches can be obtained using the analytical method.

**Figure 7:**
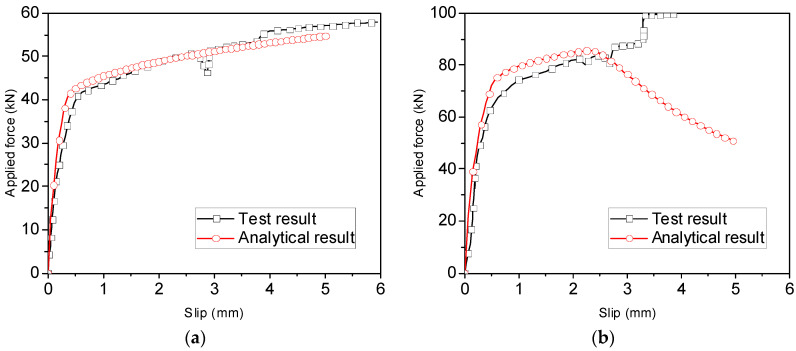
Comparison between experimental and analytical force–slip relationships of rebars with different diameters [9] for (**a**) V-1~5 and (**b**) VI-1~5.

Two more pull-out tests conducted by Ling et al. [9] were used to verify the accuracy of the analytical method, as shown in Figure 7a,b). Once again, good agreement is obtained in terms of the load–slip relationship. However, it should be pointed out that the failure mode of VI-1~5 cannot be accurately predicted using the analytical method, as shown in Figure 7b, even though the calculated load capacity of rebars is close to the experimental value. The method predicts pull-out failure of the steel rebars at the post-yield stage, whereas the rebars fractured in the tests.

**Figure 8:**
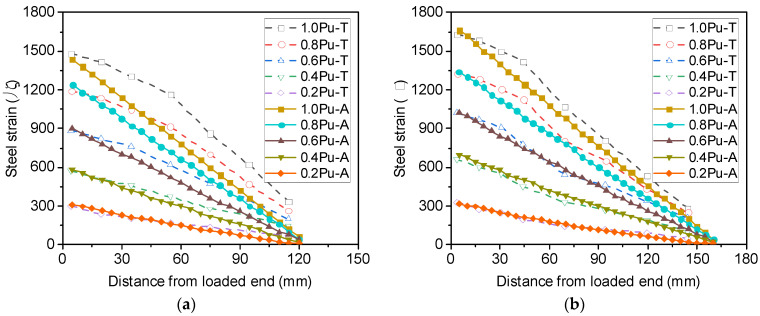

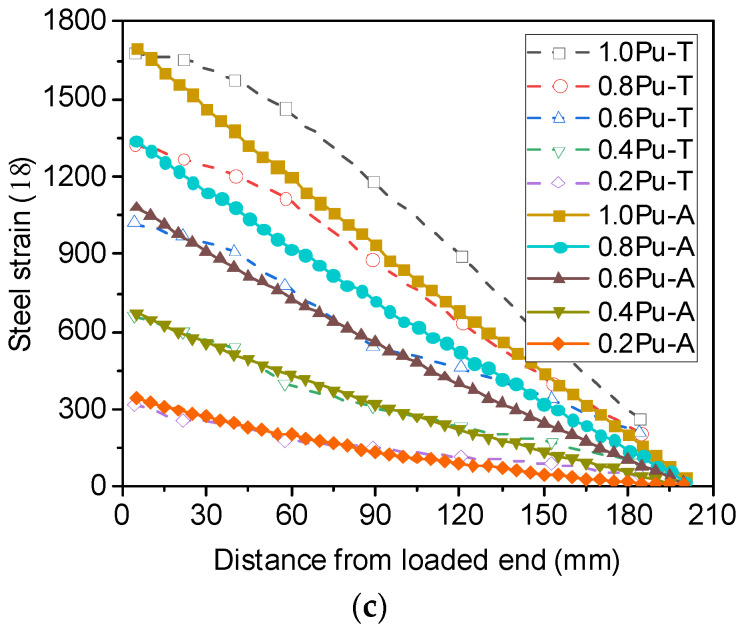
Comparison between experimental and analytical strain profiles along ribbed bars [14,15] for (**a**) S16-120-2; (**b**) S16-160-2; and (**c**) S16-200-2.

Besides the load–slip curves, the variation in steel strains along the anchorage length of the reinforcement can also be obtained using the analytical model, as shown in Figure 8. In the legend, 0.2 Pu denotes a load level of 20% of the ultimate load, and the last letters T and A represent the test and analytical results, respectively. Note that Pu in S16-200-2 was set to be the yield strength of the rebars [14]. It can be observed that the calculated strain decreases almost linearly from the loaded end to the free end at different load levels. Comparisons between the measured and calculated strain profiles of the reinforcement show that when the load level is no more than 60% of the ultimate load, the calculated strain profile agrees well with the measured one from the loaded end to the free end. However, with increasing load level, the strain profile levels off near the loaded end, and the analytical method substantially underestimates the strain in the region, particularly in S16-200-2. This phenomenon could have resulted from local bond failure near the loaded end while the load level was relatively high.

**Figure 9:**
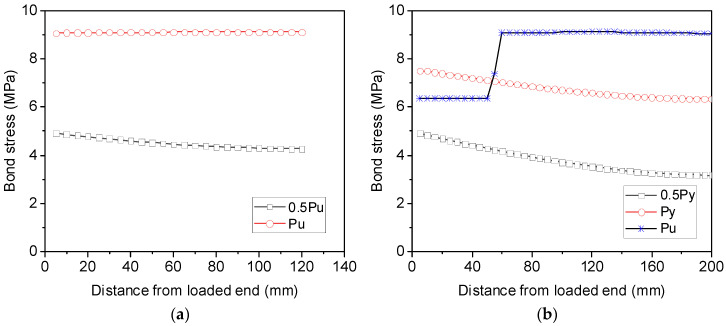
Analytical bond stresses of steel rebars along the embedment length for (**a**) S16-120-2 and (**b**) S16-200-2.

Figure 9 shows the profiles of bond stress over the anchorage length of S16-120-2 and S16-200-2. It can be observed from Figure 9a that for S16-120-2, the bond stress is not uniform when the load applied to the reinforcement is only 50% of its load capacity, with its minimum value at the free end and maximum value at the loaded end. However, nearly constant bond stress is distributed along the anchorage length once the maximum load of the reinforcement is attained. By increasing then anchorage length from 120 mm to 200 mm, the nonlinear bond stress distribution becomes more significant before the reinforcement yields at the loaded end, as shown in Figure 9b. Following yielding of the reinforcement, the bond stress drops to 6.4 MPa, but the bond stress over the elastic steel segment remains uniform. The calculated results from the analytical method agree well with the assumption made for calculating the post-yield bond stress using Equation (2).

### 3.2. Bond–Slip Behaviour of Threaded Rods

**Figure 10:**
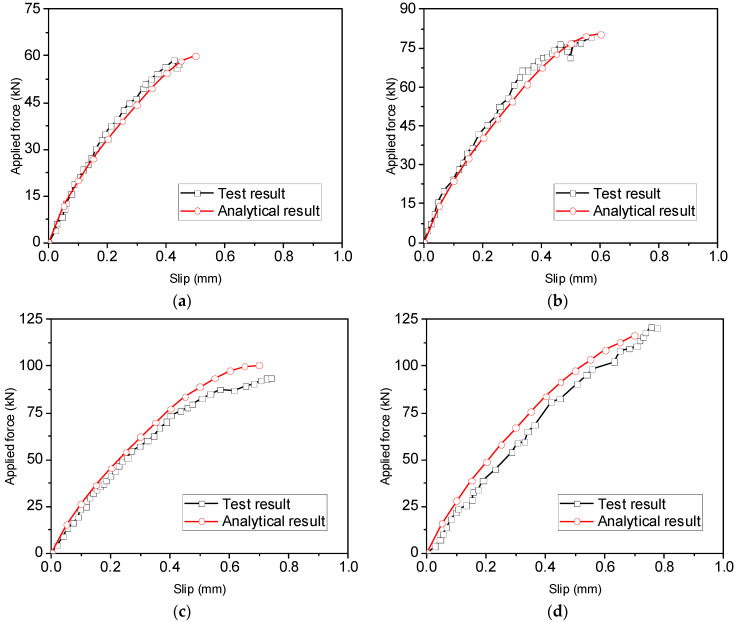
Comparison between experimental and analytical force–slip relationships of threaded rods for (**a**) R16-120-2; (**b**) R16-160-2; (**c**) R16-200-2; and (**d**) R16-240-2.

In addition to ribbed bars, the bond–slip behaviour of steel rods tested by Ling et al. [9] can also be analysed using the proposed model. Figure 10 shows a comparison between experimental and analytical force–slip relationships of steel rods. The analytical results are in good agreement with the test data in terms of the initial stiffness and load capacity of the glued-in rods. However, as the yield strength of the steel rods was substantially higher than that of the ribbed bars, all rods exhibited pull-out failure at the elastic stage, and no yielding of rods was observed, as can be found in the force–slip relationship.

**Figure 11:**
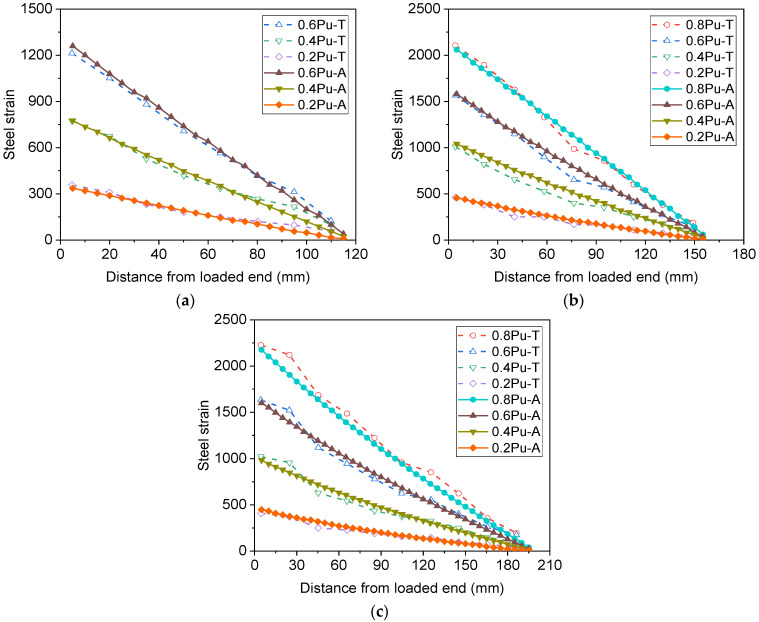
Comparison between experimental and analytical strain profiles of threaded rods for (**a**) R16-120-2; (**b**) R16-160-2; and (**c**) R16-200-2.

Figure 11 shows a comparison of the strain profiles along the anchorage length of the threaded rods. Note that nearly the same strains at the loaded end were selected from the experimental and analytical results to facilitate comparisons. As all rods were pulled out from the glulam at the elastic stage, rather linear strain profiles are obtained at different load levels, and the analytical results are in good agreement with the measured values. Nevertheless, strain profiles corresponding to the load capacity of steel rods are not included in the comparison, as the calculated strain at the loaded end is far less than the measured value when the load capacity is attained.

**Figure 12:**
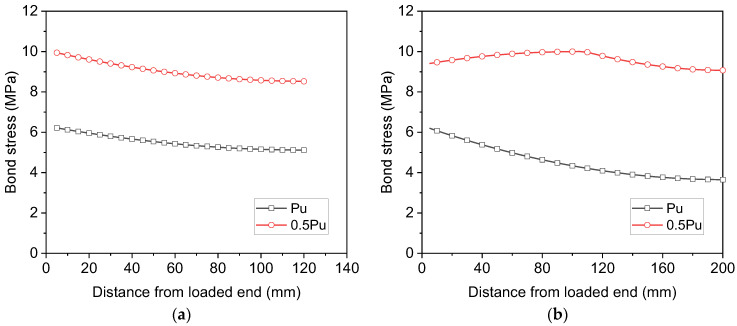
Analytical bond stresses of threaded rods along the length for (**a**) R16-120-2 and (**b**) R16-200-2.

Figure 12 shows the bond stress distribution over the length of the threaded rods. It can be observed that when the embedment length of the threaded rods is 120 mm, the bond stress distribution is similar to that of S16-120-2, as shown in Figure 12a, with the maximum bond stress at the loaded end and the minimum bond stress at the free end. As the ultimate load of R16-160-2 is greater than that of R16-120-2, the associated bond stress in the same section is also larger. Nevertheless, when the embedment length is increased to 200 mm, the bond stress distribution of R16-200-2 at the ultimate load becomes significantly different from that of S16-200-2 (see Figure 12b). As the yield strength of the threaded rods is considerably greater than that of ribbed bars, the steel rod R16-200-2 does not yield at the ultimate load, no sudden drops in the bond stress can be observed at the loaded end, and the calculated bond stress at the loaded end is slightly smaller than that in the middle of the embedment length.

## 4. Lengths for Yield and Ultimate Strengths of Rebars and Rods

Based on the calculated bond stress distribution along the anchorage length of the rebars or rods, the minimum length required for the reinforcement to develop yield strength can be calculated as follows.

(6) $$l_{y}=\frac{f_{y}d}{4\tau_{m}}$$

where $f_{y}$ is the yield strength of the reinforcement or rods, d is the diameter, and $l_{y}$ is the embedment length of the reinforcement or rods to develop yield strength.

For the mechanical and geometric properties of the steel reinforcement, rods, and glulam listed in Table 1 and Table 2, the required embedment length for reinforcement to develop yield strength can be determined as 160 mm, 10 times the rebar diameter. By contrast, the required anchorage length for steel rods to yield is increased to 356 mm, around 22.3 times the rod diameter, as the yield strength of rods is considerably greater than that of ribbed bars. Therefore, it is difficult to yield the rods in the range of anchorage length adopted in our experimental programme. 

Nevertheless, it should be pointed out that even if a longer embedment length is provided in the design, steel reinforcement may still be pulled out from the glulam, as shown in Figure 6c, leading to a gradual reduction in the applied force at the post-yield stage of reinforcement. This phenomenon can be attributed to the fact that the bond stress of the reinforcement is substantially reduced once it enters the post-yield stage, and the reduction in post-yield bond stress cannot be compensated by the increase in the elastic bond stress. Therefore, to prevent pull-out failure of the reinforcement, a longer embedment length than that calculated using Equation (7) should be used in the design.

(7) $$l_{u}=\frac{f_{y}d}{4\tau_{m}}+\frac{\left(f_{u}-\;f_{y}\right)d}{4\tau_{y}}$$

where $l_{u}$ is the required embedment length for the steel reinforcement to reach its ultimate strength; $f_{u}$ is the ultimate strength of the reinforcement; and $\tau_{y}$ is the post-yield bond stress of the reinforcement glued in glulam, calculated using Equation (2). 

As the post-yield bond strength is generally smaller than that at the elastic stage, the embedment length of the reinforcement associated with the ultimate strength is expected to be significantly longer than that corresponding to the yield strength. By using the mechanical and geometric properties in Table 1 and Table 2, the embedment length for ultimate strength can be quantified as around 298 mm, nearly 18.6 times the rebar diameter.

## 5. Comparisons with Design Equations

Even though the proposed method can be used to calculate the load resistance and slip of steel rebars and rods glued in glulam, a simplified design equation may be preferred if only the load capacity is of concern. Riberholt [3] proposed a design equation for calculating the pull-out capacity of glued-in bolts (see Equation (8)), in which the effect of anchorage length was considered. Note that the equation is only valid when the bonded length is greater than 200 mm. If the bonded length is less than or equal to 200 mm, a constant bond strength of 5 N/mm^2^ is suggested.

(8) $$f_{v,k}=\left(1/\pi \right)K_{c.\mathrm{adhesive}.k}\rho l^{-0.5}$$

where ρ is the density of timber, and $K_{c.\mathrm{ad}h\mathrm{esive}.k}$ is the fracture toughness and equal to 520 N/mm^1.5^.

The design code for timber structures in Germany [5] incorporates a value for average bond strength, as expressed in Equation (9). When the anchorage length is less than 250 mm, a constant bond strength of 4.0 MPa can be used in the design. The average bond strength is defined as a piecewise linear function of the anchorage length greater than 250 mm.

(9) $$\begin{array}{cc} f_{k1}=4.0 & l_{a}\;\leq \;250\\ f_{k1}=5.25\;-\;0.005l_{a} & 250\;<\;l_{a}\;\leq \;500\\ f_{k1}=3.5\;-\;0.0015l_{a} & 500\;<\;l_{a}\;\leq \;1000 \end{array}$$

where $f_{k1}\;$ is the average bond strength and $l_{a}$ is the anchorage length of the rebars or rods.

Steiger et al. [4] proposed an empirical equation based on test data and defined the average bond strength as a function of the slenderness ratio of rods and the density of glulam, as expressed in Equation (10).

(10) $$f_{v,0,\mathrm{mean}}=7.8{\left(\frac{\lambda }{10}\right)}^{-1/3}{\left(\frac{\rho }{480}\right)}^{0.6}$$

where $f_{v,0,\mathrm{mean}}$ is the average bond strength at the glue/timber interface and λ is the slenderness ratio in the range of 7.5 and 15, defined as the ratio of anchorage length and the diameter of the borehole.

Rossignon and Espion [16] recommended a formula for calculating the average bond strength of steel rods parallel to the grain, as expressed in Equation (11). This equation us similar to that proposed by Steiger, but only the effect of the slenderness ratio is considered in this equation.

(11) $$f_{v,0,\mathrm{mean}}=5.8{\left(\frac{\lambda }{10}\right)}^{-0.44}$$

On account of the differences in the mechanical properties of rebars and rods, the abovementioned equations may not be suitable for calculating the average bond strength of glued-in rebars. The analytical results show that when the steel reinforcement is at the elastic stage, namely, the anchorage length is less than 10 times the rebar diameter, the average bond stress can be taken as the peak bond stress in Equation (1). Otherwise, both the elastic and post-yield bond stresses have to be considered in calculating the average bond stress.

For a given anchorage length greater than 10 times the rebar diameter, Equation (12) can be used to quantify the average bond stress. In the calculation, bond stresses are assumed to be uniform over the elastic and yielded steel segments. Thus, the average bond strength depends on the yield strength and anchorage length of the steel reinforcement. By increasing the anchorage length, the average bond strength can be reduced due to yielding of the rebars near the loaded end and the resulting reduction in bond stress, as suggested by previous researchers.

(12) $$\begin{array}{cc} \tau_{a}=\tau_{m} & l\leq l_{y}\\ \tau_{a}=\frac{f_{y}d}{4l}+\frac{\tau_{y}\left(l-l_{y}\right)}{l} & l\;>\;l_{y} \end{array}$$

**Figure 13:**
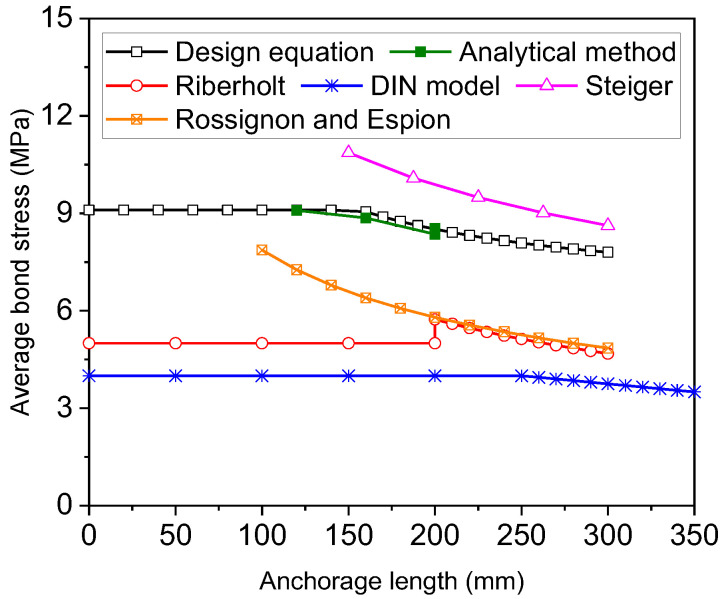
Comparison between average bond stresses of steel rebars and rods from different methods.

Figure 13 shows the relationship between the average bond stress and the anchorage length of the steel rebars. Note that the average bond strength at the glue/timber interface, calculated using Equation (11), is converted into that at the rebar/glue interface. The average bond strength is calculated using the analytical method by dividing the applied load by the area of the interface between the steel rebars and glue. As the analytical method can predict the overall load–slip curves with good accuracy, the calculated average bond strength is also close to test data. The average bond strength calculated from Equation (12) agrees well with that from the analytical model. Comparisons between the different equations suggest that the equations developed by Riberholt [3] and DIN 1052 [5] substantially underestimate the average bond strength of steel rebars. Steiger’s equation [4] predicts higher bond strengths than Equation (12). Rossignon and Espion’s equation predicts a similar variation to Steiger’s equation but significantly smaller bond strength. Moreover, the average bond strength determined using Rossignon and Espion’s equation is close to that computed using Riberholt’s equation when the anchorage length is greater than 200 mm.

Our analytical study shows that when the load capacity of glued-in rebars is attained, the reduction in average bond stress with increasing anchorage length can mainly be attributed to the yielding of rebars at the loaded end and the ensuing decrease in bond stress over the yielded steel segment. However, with regard to high-strength steel rods, it is not possible to reach yield strength with a practical range of anchorage length, and the nonlinear distribution of bond stress results from the high yield strength. Similar results can be expected if steel rebars with high yield strength of basalt fibre-reinforced polymer [17] are used. Further experimental and analytical studies are needed to explore the applicability of the proposed method to high-strength steel rebars and rods.

## 6. Conclusions

This paper describes an analytical study on bond–slip behaviour of ribbed bars and threaded rods anchored in glulam along the grain. In the study, the relationship between a local bond–slip model and global bond behaviour is established. Comparisons between experimental and analytical results show that the analytical method can be used to evaluate the bond–slip behaviour of reinforcement with good accuracy. The following conclusions can be drawn from the analytical study.

1. By using the bond–slip curves measured from tests of short rebars or rods, the load capacity, slip, and failure mode of long rebars and rods along the grain of glulam can be predicted using the analytical method with good accuracy. In the calculation, the post-yield bond stress of rebars calculated from test data is around 70% of the peak elastic bond stress.
2. The analytical results show that when the load capacity of the rebars is achieved, the bond stresses can still be assumed to be uniform over the elastic and yielded steel segments, respectively. Equations are developed for calculating the anchorage lengths of rebars associated with the yield and ultimate strengths.
3. For the given material properties in the experimental tests, the lengths for the yield and ultimate strengths of the rebars are determined to be 10 and 18.6 times the rebar diameter, respectively. Nonetheless, the required length for the yield strength of the threaded rods is 22.3 times the rod diameter as a result of its relatively high yield strength compared with the steel re-bars.
4. An equation is also derived for average bond stress over the anchorage length of the long reinforcement, which takes account of the effects of yield strength, post-yield bond stress, and anchorage length. Comparisons with existing equations of average bond strength indicate that the design equations proposed by Riberholt and DIN 1052 underestimate the average bond strength of deformed rebars, whereas Steiger’s equation yields overestimations of the average bond strength.

## Figures and Tables

**Table 1:** Properties of steel reinforcement.

Specimen ID	Type	Steel Grade	Diameter/Anchorage Length (mm)	Yield Strength (MPa)	Ultimate Strength (MPa)	Elastic Modulus (GPa)	Hardening Modulus (MPa)
S16-120-2	Ribbed bars	HRB335	16/120	362	556	2.05	1000
S16-160-2			16/160				
S16-200-2			16/200				
V-1~5	Ribbed bars	HRB335	12/180	350	571	2.06	1000
VI-1~5	Ribbed bars	HRB335	16/200	362	556	2.06	1000
R16-120-2	Steel rods	M8.8	16/120	695	842	2.01	--
R16-160-2			16/160				
R16-200-2			16/200				
R16-240-2			16/240				

**Table 2:** Properties of glulam timber parallel to grain.

Specimen ID	Compressive Strength (MPa)	Tensile Strength (MPa)	Shear Strength (MPa)
S16-120-2, S16-160-2, S16-200-2	36.0	26.1	10.3
V-1~5, VI-1~5	36.0	102.1	10.6
R16-120-2 R16-160-2 R16-200-2 R16-240-2	36.0	42.0	8.4

## Data Availability

The data presented in this study are available upon request from the corresponding author.

## References

[1] Steiger R., Serrano E., Stepinac M., Rajcic V., O’Neill C., McPolin D., Widmann R. (2015). Strengthening of timber structures with glued-in rods. Constr. Build. Mater..

[2] Xu B.H., Bouchair A., Racher P. (2012). Analytical study and finite element modelling of timber connections with glued-in rods in bending. Constr. Build. Mater..

[3] Riberholt H. Glued bolts in glulam-proposals for CIB code. Proceedings of the 21th Conference of CIB-W18.

[4] Steiger R., Gehri E., Widmann R. (2007). Pull-out strength of axially loaded steel rods bonded in glulam parallel to the grain. Mater. Struct..

[5] (2008). Entwurf, Berechnung und Bemessung von Holzbauwerken.

[6] Xu B.-H., Li D.-F., Zhao Y.-H., Bouchair A. (2020). Load-carrying capacity of timber joints with multiple glued-in steel rods loaded parallel to grain. Eng. Struct..

[7] Xu B.-H., Guo J.-H., Bouchair A. (2020). Effects of glue-line thickness and manufacturing defects on the pull-out behavior of glued-in rods. Int. J. Adhes. Adhes..

[8] Ling Z., Yang H., Liu W., Lu W., Zhou D., Wang L. (2014). Pull-out strength and bond behaviour of axially loaded rebar glued-in glulam. Constr. Build. Mater..

[9] Ling Z., Xiang Z., Liu W., Yang H., Tang J. (2019). Load-slip behaviour of glue laminated timber connections with glued-in steel rod parallel to grain. Constr. Build. Mater..

[10] Shima H., Chou L.L., Okamura H. (1987). Micro and macro models for bond in reinforced concrete. J. Fac. Eng. Univ. Tokyo.

[11] Bigaj A.J. (1995). Bond Behaviour of Deformed Bars in NSC and HSC-Experimental Study.

[12] Tlustochowicz G., Serrano E., Steiger R. (2011). State-of-the-art review on timber connections with glued-in steel rods. Mater. Struct..

[13] Kang S.B., Tan K.H. (2016). Bond-slip behaviour of deformed reinforcing bars embedded in well-confined concrete. Mag. Concr. Res..

[14] Ling Z. (2015). Bond-Anchorage and Seismic Behaviors of Glulam Joints with Glued-in Rods. Ph.D. Thesis.

[15] Ling Z., Liu W., Yang H., Chen X. (2018). Modelling of glued laminated timber joints with glued-in rod considering bond-slip location function. Eng. Struct..

[16] Rossignon A., Espion B. (2008). Experimental assessment of the pull-out strength of single rods bonded in glulam parallel to the grain. Holz Als Roh—Werkst..

[17] Yeboah D., Taylor S., McPolin D., Gilfillan R. (2013). Pull-out behaviour of axially loaded Basalt Fibre Reinforced Polymer (BFRP) rods bonded perpendicular to the grain of glulam elements. Constr. Build. Mater..

